# Ultrasound-Targeted Microbubble Destruction Alleviates Immunosuppression Induced by CD71^+^ Erythroid Progenitor Cells and Promotes PDL-1 Blockade Immunotherapy in the Lewis Lung Cancer Model

**DOI:** 10.3389/fonc.2021.768222

**Published:** 2021-10-22

**Authors:** Xi Tan, Cuo Yi, Yi Zhang, Najiao Tang, Yali Xu, Zheng Liu

**Affiliations:** Department of Ultrasound, Xinqiao Hospital, The Second Hospital of The Army Medical University, Chongqing, China

**Keywords:** CD71^+^ erythroid progenitor cells, erythroid progenitor cells, ultrasound-targeted microbubble destruction, systematic immunity, sonoporation

## Abstract

The CD71^+^ erythroid progenitor cells (CECs) exhibit distinctive immunosuppressive properties and regulate antitumor immunity to enable tumor growth. We presented a novel and non-invasive approach to improving immunity by targeting the splenic CECs *via* sonoporation generated by ultrasound-targeted microbubble destruction (UTMD). The systematic immunity enhanced by the reduction of PDL-1-expressing CECs also benefits the PDL-1 blockade therapy. In the Lewis lung cancer (LLC) model, the study group was treated by UTMD for 10 min at the splenic area with or without anti-mouse PDL-1 intraperitoneal injection. The frequency of splenic CEC, lymphocyte, and cytokine production was analyzed by flow cytometry. Serum interleukin-2 (IL-2) was tested by ELISA. Tumor volume was evaluated by two-dimensional ultrasound. The UTMD treatment consisted of ultrasound sonication and Sonazoid™ microbubble injection through the caudal vein. The mechanic index (MI) of ultrasound was set between 0.98 and 1.03. The results showed a significant reduction of splenic CECs and increased frequency of CD8^+^ T cells treated by UTMD treatment in the late-stage tumor. Tumor growth could be inhibited by UTMD combined with PDL-1 blockade therapy. The frequencies of interferon-γ (IFN-γ) producing CD8^+^ and CD4^+^ T cells were significantly increased after being treated by the combination of UTMD and PDL-1 blockade, while the reactive oxygen species (ROS) production and the fraction of the TGF-β-producing CD11b^+^ cells were significantly decreased. These preliminary findings suggest that UTMD enhances immune response and facilitates PDL-1 blockade therapy by targeting immunosuppressive CECs in the spleen. Our study provides new aspects and possibilities for treating cancer-related infection and tumor control in oncology.

## 1 Introduction

Cancer immunotherapy has revolutionized therapeutic strategies in clinical oncology over the last decade. Modulating the immune system through immune checkpoint inhibitors has led to durable remissions across various cancers, but the efficacy remains limited (1). To overcome the challenges, massive studies have focused heavily on local immune responses in the tumor microenvironment (TME). Emerging evidence suggests that immunotherapy drives new immune responses rather than the reinvigoration of preexisting immune responses. The systemic immunity in cancer beyond the TME is essential for effective immune response toward immunotherapy, and the tumor immune macroenvironment is remarkably plastic (2–4). Recently, immature red blood cells called CD71^+^ erythroid progenitor cells (CECs) or erythroid progenitor cells (EPCs) were identified as regulators of the immune response in cancer (5, 6). The CD71/Ter119 combination-marked CECs reflect the dynamic maturation of red blood cells, which define the nucleated erythrocytes (7). CECs expand in the enlarged spleen due to dysregulated erythropoiesis and potently suppress the systematic immune response. Suppressed immune response induces tumor immune evasion to promote tumor growth and leads to increased susceptibility to infections. Extensive studies revealed its neglected immunomodulatory properties under other different physiological and pathological conditions, too. CEC is heterogeneous, which could be induced by tumor, injury (8), pregnancy (9), systemic inflammation (10), HIV infection (11), and even COVID-19 (12, 13). All phenotypes have immunomodulatory functions. In tumor-bearing mice, the proliferation and cytotoxicity of CD8^+^ T cells, the proliferation of CD4^+^ T cells, and TH1 differentiation were inhibited by CEC. During tumorigenesis, splenomegaly was observed at the later stages. The spleen size increase was mainly attributed to CECs (14). It suppresses the immune response by inhibiting the production of interferon-γ (IFN-γ) *via* soluble cytokines such as TGF-β. Interestingly, CEC also modulates T cells through direct cell–cell interaction *via* PDL-1/PD-1 pathway and expresses genes encoding immune checkpoint molecules (15), so we assume that the absence of CEC not only increases the production of immunomodulated cytokines but also could act as a checkpoint blockade, and the immune checkpoint inhibitors may partially diminish the tumor-promoting effects of CECs as well. Therefore, CECs could be a new target to restore peripheral immune perturbation in cancer and enhance immunotherapy’s efficiency to inhibit tumor growth.

When ultrasound-excited microbubbles (MBs) (preexisting MBs *in vivo* or injected MBs) resonate around biological barriers, they will release a series of mechanical effects such as microstreaming, microjets, and shock waves. This process is called ultrasonic cavitation (16). During the activity of cavitation, ultrasound-activated mechanical force generated by the vibrating MBs forms perforation on the cell membrane, which is known as “sonoporation.” The biological effect of sonoporation usually depends on the acoustic pressure amplitude, the concentration of MBs, and the properties of targeted cells (15–17). Ultrasound at low acoustic pressure (<300 kPa) produces reversible pores on the cell membrane. The permeability of the cell membrane was increased without endangering the cell viability (16–18). Therefore, recent studies have extensively focused on drug/gene delivery by increasing local drug concentration to achieve therapeutic effects (19–21). The high acoustic pressure (>300 kPa) ultrasound often forms lethal pores on the cell membrane or blood vessels, physically destroying the survival components to perform cell killing, which is generally used for tumor neovascularization in current studies (22, 23). We noticed that both therapeutic ultrasound and tumor immunology fields had focused locally in the TME, while cancer is a systematic disease. Treatments for improving peripheral immunity may have unexpected results. Currently, no effective treatment has been reported against the immunosuppressive CECs. We presumed that the sonoporation effect stimulated by high-acoustic-pressure ultrasound might mechanically attack the CECs accumulated in the enlarged spleen, enhance the systematic immunity, and even inhibit tumor growth based upon the adjustable feature of sonoporation.

To testify that our hypothesis that ultrasound-targeted MB destruction (UTMD) could modulate the suppressed immune response and promote PDL-1 blockade therapy to inhibit tumor progression by striking tumor-induced CEC in the spleen, the Lewis lung cancer (LLC) animal model was built. The tumor-bearing mouse was treated with a diagnostic–therapeutic function-combined ultrasound apparatus in mouse spleen along with an injection of Sonazoid™ MBs through the caudal vein. As we predicted, the results showed that the frequency of CEC was significantly reduced. The immunocompetent lymphocytes were significantly increased in the treated group. The tumor growth curve showed no difference merely treated by UTMD. To explore more possibilities, we combined UTMD and the PDL-1 blockade therapy, and the result showed that the combination therapy could inhibit tumor growth compared with the control group after three-times combination treatments. Cytokine production was analyzed by flow cytometry, and the mechanism of the antitumor effect involved increasing production of IFN-γ in CD4^+^ and CD8^+^ T cells and the decreasing of reactive oxygen species (ROS) and TGF-β CD11b ^+^ cells in our study. In summary, our finding suggested that UTMD enhances the immunity in the late-stage tumor by targeting the CEC, and the combination of UTMD and PDL-1/PD-1 blockade therapy will be a promising therapeutic strategy for cancer treatment.

## 2 Method

### 2.1 Cell Line and Animal Model

LLC cells were purchased from the America Type Culture Collection (ATCC), cultured in Dulbecco’s modified Eagle’s medium (DMEM)/H (HyClone), supplemented with 10% fetal bovine serum (FBS) (Gibco, USA) and 1% penicillin–streptomycin (HyClone), and incubated at an atmosphere of 37°C with 5% CO_2_/95% air. Cell lines are routinely validated and without contamination. Both male and female C57BCL/6 mice were acquired from The Jackson Laboratory (USA). LLC cells (2 × 10^6^) were resuspended in 200 μl of phosphate-buffered (PBS; HyClone) for animal injection. The cell resuspension was subcutaneously injected into the right flank of the C57 mice at 4 to 6 weeks, and at 21 days after inoculation, the late-stage tumor is defined. The largest diameter of tumors smaller than 15 mm or more extensive than 20 mm was excluded. The cell resuspension was subcutaneously injected on the back for convenient measurements to observe the impact on the tumor growth. Seven to 9 weeks of regular C57 mice were also included. All procedures were approved by the Laboratory Welfare and Ethics Committee of The Army Medical University, in line with animal ethics and animal welfare requirements.

### 2.2 Ultrasound-Targeted Microbubble Destruction Protocol

#### 2.2.1 Ultrasound Equipment and Acoustic Parameters

The ultrasound equipment was VINNO 70 (VINNO Technology Co. Ltd, Suzhou, China) with a high-frequency linear array probe (X4-12L). VINNO 70 is a diagnostic–therapeutic function-combined apparatus. The diagnostic part is for the sonography, including two-dimensional and contrast-enhanced imaging. The therapeutic part relied on the modified Vflash mode imposed on low MI (0.04) contrast-enhanced ultrasonography. Vflash mode emits intermittent impulses with a region of interest (ROI) on the spleen, providing adjustable parameters for various ultrasound cavitation conditions. Vflash treatment parameter setting was as follows: frequency 5 MHz, pulse length 6.5 cycles, pulse repetition frequency (PRF) 500 Hz, transmitting and intermittent time 0.1 and 0.1 s, acoustic power 80%, MI 0.98 to 1.03, and duration 600 s.

**Figure 1:**
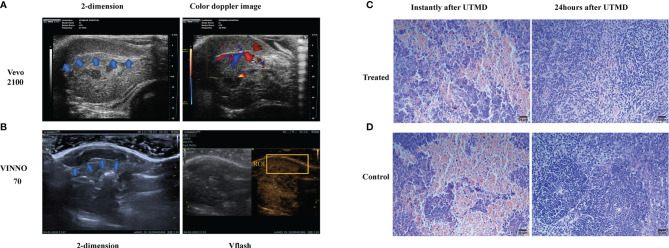
The sonography and microscopy observation of the tumor-bearing mouse spleen. **(A)** The two-dimensional and color Doppler flow images of the tumor-bearing mouse spleen (shown in blue arrows) with a small animal ultrasound system (Vevo2100, USA). **(B)** The two-dimensional and contrast-enhanced ultrasound sonography on the diagnostic–therapeutic ultrasonic apparatus VINNO 70 (VINNO Technology Co. Ltd, Suzhou, China). The region of interest (ROI) is shown by the orange box. Respective H&E-dyed slice observation of the tumor-bearing mice instantly and 24 h after ultrasound-targeted microbubble destruction (UTMD) treatment in treated **(C)** and control **(D)** groups.

The sonography of the spleen was obtained in an advanced LLC mouse model. The spleen of the tumor-bearing mice was located by a small animal ultrasound system (Vevo2100, USA). The location of the spleen is relatively superficial (**Figure 1A**), which is less than 1 mm from the skin, so the acoustic energy can fully transmit to the spleen area. The treatment was utilized by the Vflash mode of VINNO 70, which was activated on a background of contrast-enhanced ultrasound mode (MI = 0.04). ROI was set to cover the rim of the spleen (**Figure 1B**), and the range of ROI was 2.0 cm × 1.5 cm.

#### 2.2.2 Microbubble Preparation

The size distribution and concentration of the commercial perfluorobutane microsphere (Sonazoid™, GE Healthcare AS, Norway) are as follows: the median diameter is approximately 2.6 μm, less than 0.1% larger than 7 μm, and the bubble concentration is 1.2 × 10^8^/ml (24). Sonazoid of 16 μl was dissolved/reconstituted with 4 ml of sterile saline in a liquid transfer kit that came with the package. Microsphere suspension of 0.01 ml was diluted into 1 ml of saline. Diluted MB of 0.1 ml was injected at the first 100 s, 0.02 ml per 100 s for three times, and 0.02 ml per 50 s at the rest of the treatment. The total treatment was 600 s, and the total amount of diluted MB was 0.24 ml. This unique injection pattern depends on the phenomenon we observed at the pre-experimental stage. At a later period of the treatment, the MBs seem to metabolize more rapidly. The control group was anesthetized and injected the same amount of saline but without sonication.

#### 2.2.3 Treatment Procedures and Tumor Volume Measurements

During the treatment, the C57 mice were put to a left lateral position. The ROI was circling the spleen on the splenic axis under Vflash mode. The probe was kept steady by the operator. All the mice that went through ultrasound treatment tolerated the procedure and used the same parameters. Mice undergoing blockade therapy were intraperitoneally injected with the PDL-1 antibody (BE0101, Bio-X-cell, USA) of 200 µg at the preset time points. The number of mice used to obtain the data in each experiment was different. The tumor size was measured by two-dimensional ultrasound at different preset time points. The tumor volume was calculated by the following formula: V = (π/6 × length × width × height).

### 2.3 Flow Cytometry

Twenty-four hours after the ultrasound treatment, the spleen of the C57 tumor-bearing mouse was made into a single-cell suspension. Antibody staining was performed in PBS containing 2% FBS (wt/vol). Splenocytes were first stimulated with Cell Stimulation Cocktail (plus protein transport inhibitors) (00-4975-93, eBioscience, USA) for 5 h at 37°C to analyze cytokine production. According to the manufacturer’s instructions, intracellular cytokine staining was performed after cell surface staining, with a Cytofix/Cytoperm Fixation/Permeabilization kit (554714, BD Biosciences, USA). Samples were collected with the fluorescence-activated cell sorting (FACS) Canto system (BD Biosciences) and analyzed with FlowJo software. *Cell surface antibodies*: PerCP/Cyanine5.5 anti-mouse/human CD11b (101228, BioLegend, USA), PE anti-mouse/human CD45R/B220 (103208, BioLegend), fluorescein isothiocyanate (FITC) anti-mouse CD3 (100204, BioLegend), PE anti-mouse CD4 (100408, BioLegend), PE/Cy7 anti-mouse CD8a (100722, BioLegend), FITC anti-mouse TER-119/erythroid cells (116206, BioLegend), and PE/Cy7 anti-mouse CD71 (113812, BioLegend). *Intracellular antibodies*: Pacific Blue™ anti-mouse TNF-α (502902, eBioscience), APC anti-mouse LAP TGF-β1 (141406), Pacific Blue™ anti-human/mouse Granzyme B (515408), Human/Mouse Arginase 1/ARG1 PE-conjugated (IC5868P, R&D Systems), APC Rat Anti-Mouse IFN-γ (554413, BD Pharmingen™). Live and death differentiation was determined by eFluor 780 (65-0865-14, eBioscience). The mean fluorescence intensity (MFI) of ROS also tested by flow cytometry using a ROS detection kit (S0033, Beyotime).

### 2.4 Enzyme-Linked Immunosorbent Assay

Twenty-four hours after the ultrasound treatment, the tumor-bearing mice were sacrificed, and 100 µl of blood was collected from the heart. After clotting and centrifuging, the serum was obtained. IL-2 levels of the serum were measured by a sandwich ELISA using a reagent kit (ab223588, Abcam, USA) according to the manufacturer’s instructions. The difference between the color of the analyte and standard is measured by a microplate reader (Thermo Scientific Varioskan™, USA), and the enzyme activity curve is drawn to calculate the concentration of the analyte.

### 2.5 Pathology

The spleen specimen of the tumor-bearing mice was harvested after UTMD treatment 24 h or instantly after UTMD, embedded in paraffin, sectioned in 3 μm, stained with H&E, and then observed under 100× light microscope.

### 2.6 RNA-seq

Total RNAs of the tumor-bearing mouse spleen were extracted and exposed to high-throughput sequencing. Bowtie2 (25) is utilized to map the clean reads to the reference gene sequence (transcriptome) and then used RSEM (26) to calculate the gene expression level of each sample. Differentially expressed genes (DEGs) among groups were analyzed by DEseq2 (27) based on the principle of the negative binomial distribution. Gene Ontology (GO) enrichment analysis and Kyoto Encyclopedia of Genes and Genomes (KEGG) pathway enrichment analysis were used to explore the biological function of genes. GO described the molecular function of the gene, the cellular component, and the biological process involved. KEGG pathway-based analysis helps further understand the biological function of genes. Pathway significant enrichment determines the essential biochemical metabolic pathways and signal transduction pathways involved in candidate genes. According to the KEGG pathway and GO annotation classification, to perform hierarchical clustering analysis and calculate the *p*-value, and the Q value was obtained by false discovery rate (FDR) correction of *p*-value. Generally, the function of Q value ≤0.05 is regarded as a significant enrichment.

### 2.7 Statistical Analysis

Data are presented as mean ± s.d. and analyzed with Prism 8.0 (GraphPad). Statistical comparisons between the study and control groups were analyzed by independent-sample t-tests and non-parametric tests. Two-tailed unpaired Student’s t-tests with 95% confidence intervals were used to calculate all *p*-values. Repeated-measures analysis of variance (RMANOVA) was performed with SPSS 19.0 software to compare the tumor growth curve among groups. Values of *p* < 0.05 were considered statistically significant.

## 3 Results

### 3.1 Microscopy Observation of the Spleen Showed No Pathological Damages in the Advanced Lewis Lung Cancer Mouse Model After Being Treated by Ultrasound-Targeted Microbubble Destruction

To testify to the safety of ultrasound treatment, we observed the H&E-dyed slices under microscopy, and compared with the control group, there was no sign of bleeding, edema, apoptosis, or inflammation; karyopyknosis, karyolysis, and karyorrhexis had been detected after sonication. The safety of sonication in the spleen was testified at two different time points, after the sonication procedure instantly and 24 h later. The H&E-dyed slice of the tumor-bearing mouse spleen showed no bleeding, edema, apoptosis, or inflammation (**Figures 1C, D**).

### 3.2 The Frequency of CD71^+^ Erythroid Progenitor Cells Reduced While CD8^+^ T Cells Increased Significantly in the Advanced Lewis Lung Cancer Mouse Model After Ultrasound-Targeted Microbubble Destruction Treatment

**Figure 2:**
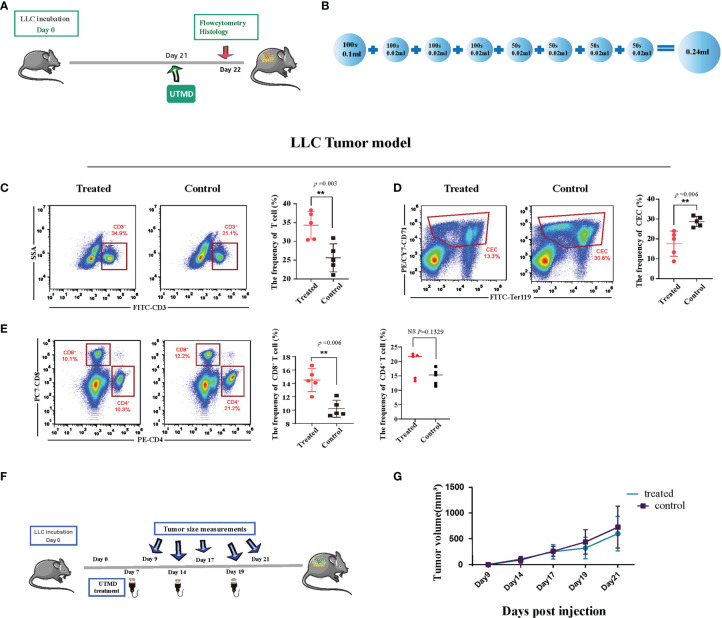
Ultrasound-targeted microbubble destruction (UTMD) alleviates immunosuppression in the late-stage tumor. **(A)** Timeline of the treatment protocol and group setting. Twenty-one days after incubation, the tumor-bearing mice in the study group received ultrasound treatment. The control group received no treatment. **(B)** The pattern of microbubble injection. A low dose of microbubble was continuously injected throughout the whole process. The total injected volume of diluted microbubble was 0.24 ml for 600 s. Tumor size measurement and treatment utilization distribution. **(C–E)** Representative flow cytometry dot plots (left) and cumulative composite data (right) of the frequency of T cells, CD8^+^ T cells and CD4^+^ T cells, and CD71^+^ erythroid progenitor cells (CECs) in the spleen of Lewis lung cancer (LLC) tumor-bearing mice. Each point represents data from an individual mouse (n = 5). **(F)** The time-point distribution of multiple treatments and tumor measurements. **(G)** The curve line of tumor growth after ultrasound treatments (n = 8). There is no significant difference in tumor volume at the start point between the two groups. The tumor growth curve also showed no difference. **P < 0.01, NS, no significance.

**Figure 3:**
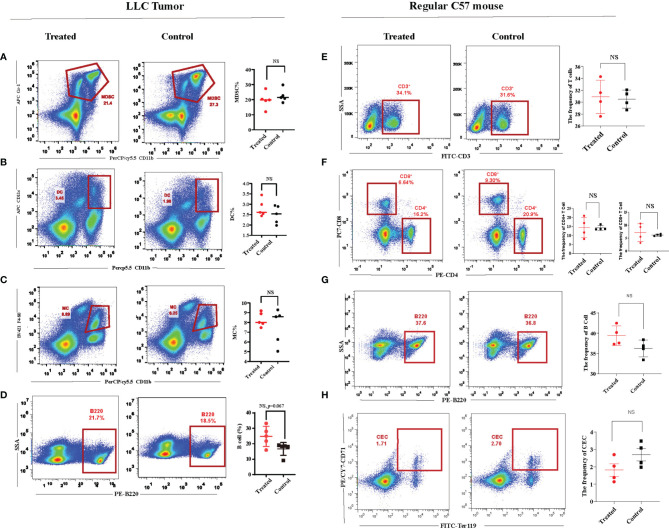
Immunity of the regular C57 mice could not be affected by ultrasound-targeted microbubble destruction (UTMD). **(A–D)** Representative flow cytometry dot plots and cumulative composite data of the frequency of dendritic cells, microphages, myeloid-derived suppressor cells (MDSCs), and B cells in the spleen of the tumor-bearing mice. Each point represents data from an individual mouse (n = 5). **(E–H)** Representative flow cytometry dot plots and cumulative composite data of the frequency of T cells, CD8^+^ T cells and CD4^+^ T cells, CD71^+^ erythroid progenitor cells (CECs), and B cells in the spleen of the regular C57 mice. Each point represents data from an individual mouse (n = 4). NS, no significance.

With the ultrasound sonication on the spleen, the protocol demonstrated (**Figure 2A**) a unique injection pattern of MB (**Figure 2B**) through the caudal vein. Twenty-four hours after UTMD treatment, we analyzed the spleen single-cell suspension of the tumor-bearing mice with flow cytometry. After excluding doublets and larger aggregates, DAPI-positive cells, nuclei, and debris, CD71^+^TER119^+^ CECs were gated for further analysis. The result showed that the percentage of splenic CECs in the spleen of the tumor-bearing mice was significantly decreased (*p* = 0.006) (**Figure 2D**). The frequency of T cells was increased (*p* = 0.003) (**Figure 2C**), and then the frequency of CD4^+^ and CD8^+^ T cells was gated and analyzed. The CD8^+^ T cells were significantly increased (*p* = 0.0057), while CD4^+^ showed no difference (**Figure 2E**). To testify whether the mere UTMD treatment could affect tumor growth, we justified the protocol. UTMD treatments were performed three times, on day 7, day 14, and day 19 after LLC incubation in the study group (**Figure 2F**). Tumor size was measured simultaneously in the study group and control group at five different time points with two-dimensional ultrasound (VINNO 70) by the method described before. Then the data were applied to draw tumor growth curves and were analyzed by SPSS. There was no significant difference in tumor volume changes between the two groups (**Figure 2G**). Moreover, we also evaluated the frequency of dendritic cells (DCs), macrophages (MCs), myeloid-derived suppressor cells (MDSCs), and B cells in the spleen of the tumor-bearing mice. However, they all showed no differences (**Figures 3A**–**D**). Additionally, we put the regular C57 mice through the same protocol. There are no significant differences shown in T cells nor CECs (**Figures 3E**–**H**).

### 3.3 The Combination of Ultrasound-Targeted Microbubble Destruction and PDL-1 Blockade Therapy Inhibited Tumor Progression

**Figure 4:**
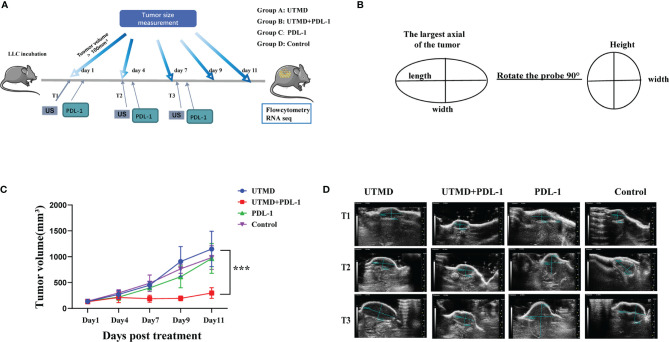
Tumor progression inhibited by the combination of ultrasound-targeted microbubble destruction (UTMD) and PDL-1 blockade therapy. **(A)** The timeline of treatments and tumor measurements with different group settings. Groups A and B received the UTMD treatment. Groups B and C received PDL-1 blockade therapy. **(B)** The illustration of tumor volume measurement with two-dimensional ultrasound. **(C)** The curve line of tumor growth in a different group setting (n = 5). **(D)** The representative two-dimensional ultrasound images of tumor size in each group by the time of the first (T1), second (T2), and third (T3) treatments. ***P < 0.001.

Since UTMD alone could not affect tumor growth, we set out to explore more possibilities. The UTMD could affect the frequency of CEC, which expresses PD-1/PDL-1. We deduced that reducing CEC introduced by UTMD could kill two birds at once, enhancing immunity and inhibiting the PD-1/PDL-1 immune checkpoint. UTMD and PDL-1 blockade therapy could probably be collaborative. We established a new protocol to determine whether UTMD combined with PDL-1 blockade could be more effective (**Figure 4A**). The starting point of treatment was when the tumor size was over 100 mm^3^, which was defined as day 0. The ultrasound treatment was utilized three times every 3 days, defined as T1, T2, and T3. The PDL-1 blockade antibody was injected intraperitoneally on the following day. The tumor volume of each group was measured at five different time points with the high-resolution ultrasound system (VEVO 2000) on day 0, day 3, day 7, day 10, and day 12. Twenty mice are divided into four groups: group A treated with ultrasound, group C received PDL-1 blockade therapy, group B received the combination therapy, and group D served as control. All the tumors were measured with the same method as demonstrated (**Figure 4B**). The largest axial of the tumor was the length. The width was vertical to the length; then rotate the probe 90° to acquire the height, which is vertical to the width. The volume was calculated by the formula V = (π/6 × length × width × height). The starting volume of the tumor was 135.98 ± 14.117 mm^3^. After being analyzed with SPSS 19.0, the tumor growth curve showed significant differences between group B and any other groups (*p* < 0.001), but no difference has been demonstrated among them (**Figure 4C**). Two-dimensional ultrasound monitors the tumor size dynamically and more accurately than traditional measurement by a vernier caliper (**Figure 4D**).

### 3.4 Ultrasound-Targeted Microbubble Destruction and PDL-1 Combination Therapy Is Effective Through IFN-γ, Especially CD8^+^ T Cell-Producing IFN-γ

**Figure 5:**
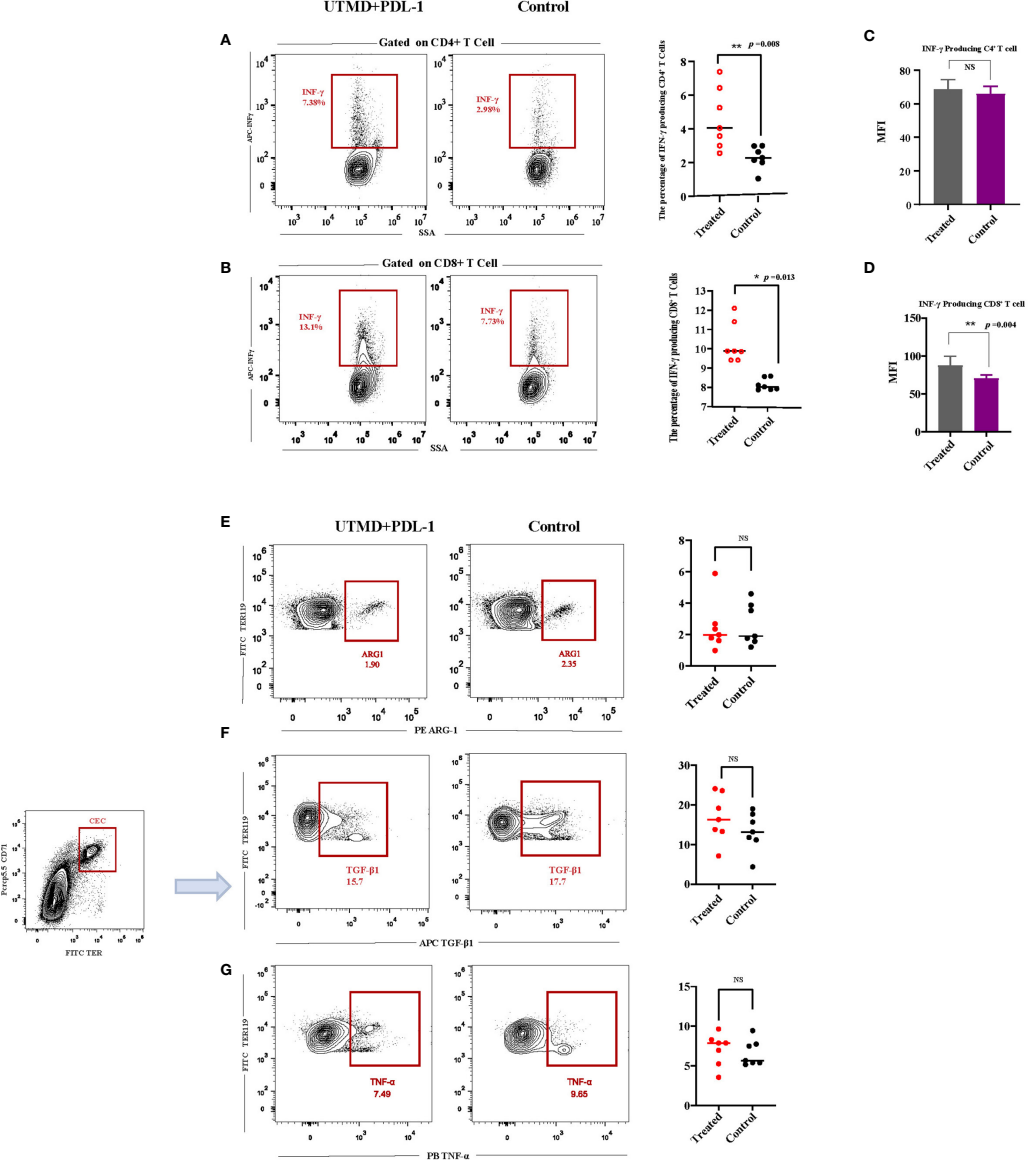
Interferon-γ (IFN-γ) and CD71^+^ erythroid progenitor cell (CEC) secreted cytokine productions after ultrasound-targeted microbubble destruction (UTMD) and PDL-1 combination therapy. **(A, B)** Representative flow cytometry contour plot and cumulative composite data of the frequencies of IFN-γ-producing CD4^+^ and CD8^+^ T cells. **(C, D)** Mean fluorescence intensity (MFI) of CD4^+^ and CD8^+^ T cells producing IFN-γ. **(E–G)** Representative flow cytometry contour plot of the relative fractions of arginase-1-, TNF-α-, and TGF-β-producing CECs, analyzed by intracellular cytokine staining. Each point represents data from an individual mouse (n = 7). *P < 0.05, **P < 0.01, NS, no significance.

**Figure 6:**
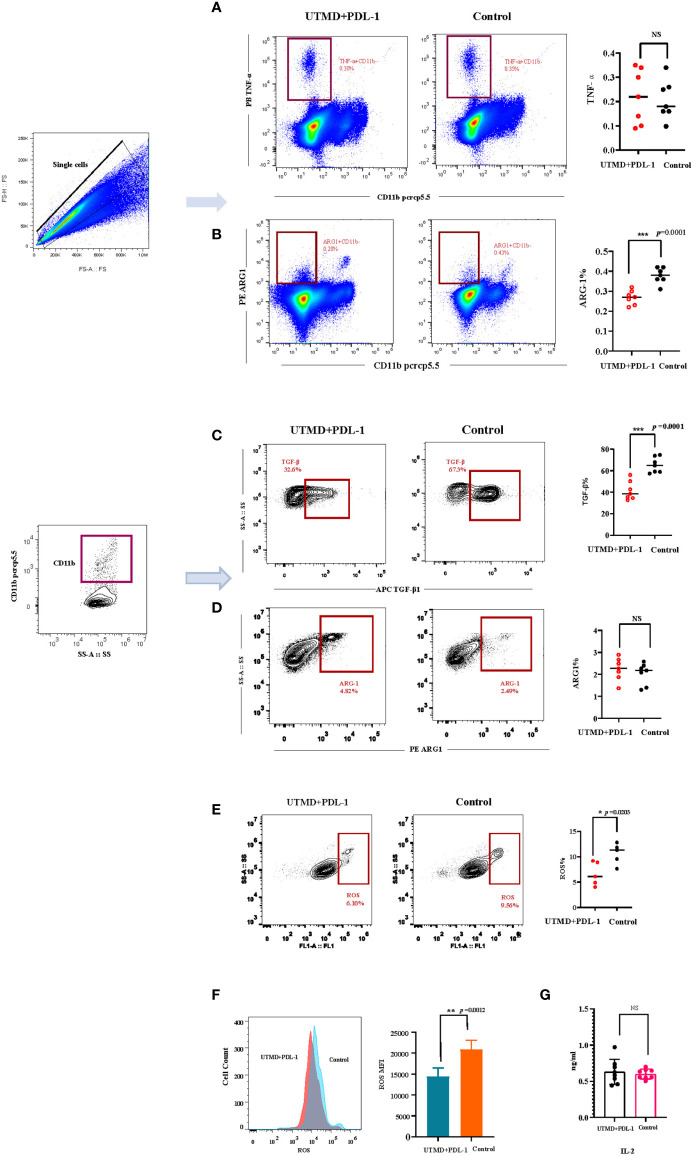
The relative fractions of arginase-1- and TGF-β-producing CD11b-negative and CD11b-positive cells. **(A, B)** Representative flow cytometry contour plot and cumulative composite data of the frequencies of TGF-β- and arginase-1-producing CD11b^+^ cells (n = 7). **(C, D)** Representative flow cytometry dot plots and cumulative composite data of the frequencies of TNF-α- and arginase-1-producing CD11b^−^ cells (n = 7). **(E)** Representative flow cytometry contour plot and cumulative composite data of the reactive oxygen species (ROS)-producing cells (n = 5). **(F)** Representative flow cytometry histogram of ROS and the cumulative composite data of mean fluorescence intensity (MFI). **(G)** The serum IL-2 (n = 8) was detected by ELISA. Each point represents data from an individual mouse. *P < 0.05, **P < 0.01, ***P < 0.001, NS, no significance.

To investigate the mechanism of ultrasound and PDL-1 combination therapy inhibiting tumor growth, the combination treatment was implemented three times as described (**Figure 3A**). According to the tumor growth curve, we analyzed the cytokine production in the splenocytes by flow cytometry between the combination of UTMD and PDL-1 blockade therapy and control groups. The results showed that the frequencies of IFN-γ-producing CD8^+^ T cells (*p* = 0.0134) and CD4^+^ T cells (*p* = 0.0082) were significantly increased (**Figures 5A, B**) after the combination therapy. Interestingly, the MFI of IFN-γ in CD8^+^ T cells (*p* = 0.0037) was increased significantly but not in CD4^+^ T cells (**Figures 5C, D**), which means that the CD8^+^ T cell-producing IFN-γ was more potent than the CD4^+^ T cell produced. Somewhat to our surprise, the relative fractions of arginase-1, TNF-α, and TGF-β-producing CECs showed no differences between the two groups (**Figures 5E**–**G**). Then we noticed a morphologically obvious subset of cells, the TNF-α-producing CD11b-negative cells. We gated out this population and analyzed the frequency of this population of cells. The cumulative composite data showed no difference between the two groups (**Figure 6A**). However, the frequency of the arginase-1-producing CD11b^−^ cells was significantly decreased (**Figure 6B**). In addition, the frequency of TGF-β-producing CD11b^+^ cells was significantly decreased (*p* = 0.0001) (**Figure 6C**), whereas no difference in arginase-1-producing CD11b^+^ cells (**Figure 6D**). The fraction of TNF-α-producing CD11b^+^ cells and TGF-β-producing CD11b^−^ cells was meager. Considering that the tumor-induced CEC also modulates the immune response through ROS, we analyzed the ROS production of the splenocytes. The frequency of the ROS-producing splenocytes cells was significantly reduced after UTMD and PDL-1 combination therapy compared with the control group (*p* = 0.0203) (**Figure 6E**), as well as the MFI of ROS (*p* = 0.0012) (**Figure 6F**). The serum IL-2 levels that were detected by ELISA showed no differences (**Figure 6G**). These results demonstrated that IFN-γ, especially CD8^+^ T cell-producing IFN-γ, plays an important role in the UTMD and PDL-1 combination treatment. The combination therapy mainly modulates cytokine production of CD11b^+^ and CD11b^−^ cells rather than the CECs.

### 3.5 Ultrasound-Targeted Microbubble Destruction and PDL-1 Blockade Combination Therapy Modulates the Expression of Genes Associated With Immune and Membrane Activity

**Figure 7:**
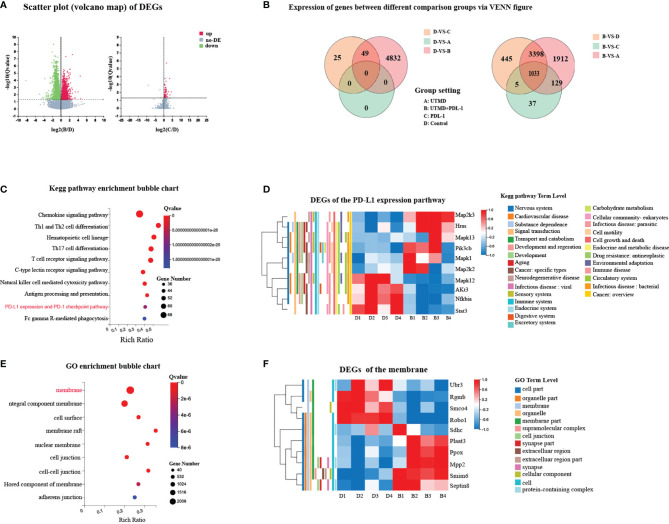
Different expression of genes between groups associated with immune activities and membrane. **(A)** Scatter plot of DEGs. The x-axis represents the fold change of the difference after conversion to log2, and the y-axis represents the significance value after conversion to-log10. Red represents DEG upregulated; green represents DEG downregulated. **(B)** Differentially expressed genes (DEGs) between different comparison groups via VENN figure. Each circle represents a group of gene sets. **(C)** Represented Kyoto Encyclopedia of Genes and Genomes (KEGG) pathway enrichment bubble chart of DEGs between groups B and D. **(D)** Heat map of representative DEGs between groups B and D regarding PDL-1 expression pathway with annotation. **(E)** Represented Gene Ontology (GO) enrichment bubble chart of DEGs between groups B and D. **(F)** Heat map of representative DEGs between groups B and D with annotation. Each column represents data from an individual mouse.

We implemented transcriptome sequencing (RNA-seq) for further exploring the mechanism of UTMD-induced immunity enhancement. The transcriptome of each group (**Figure 3A**) was analyzed to the reference transcriptome, and the gene expression level was calculated. The combination therapy group clearly showed a different gene expression profile compared with other groups, demonstrated by the VENN figure (**Figure 7A**). The scatter plot presented that DEGs between the UTMD and PDL-1 blockade combination therapy group and the control group have enormously outnumbered them between the PDL-1 blockade-treated group and the control group (**Figure 7B**); the UTMD-treated group and the control group showed no DEGs, which means that the DEGs between these two groups were below the threshold; on the other hand, it means that the DEGs between group A and D were far less than those between the other groups. The primary biological function of candidate genes was determined through the GO and KEGG pathway enrichment analysis. The Q value (corrected *p*-value) ≤0.05 was defined as significantly enriched candidate genes. A total of 4,881 DEGs were revealed between groups B and D (fold change ≥ 2 and FDR ≤ 0.001). According to KEGG pathway enrichment analysis, there are highly expressed DEG biological functions regarding immune activities, such as antigen processing and presentation, natural killer cell-mediated cytotoxicity pathway, T-cell receptor signaling pathway, and the PD-1/PDL-1 pathway (**Figure 7C**). The transcriptome profile of the UTMD and PDL-1 blockade combination therapy-treated group was separate from that of the control group (**Figure 7D**). GO analysis demonstrated the candidate genes of the cellular component involved in these DEGs (**Figure 7E**). Highly expressed genes of the membrane are shown in a heat map (**Figure 7F**). These results revealed that the UTMD-induced sonoporation could assist PDL-1 blockade therapy in enhancing the efficacy against tumor progression in our study.

## 4 Discussion

Overall, our study originally presented UTMD to modulate the immunity outside TME by targeting the immunosuppressive splenic CECs. UTMD was performed by diagnostic ultrasound equipment with the improved cavitation feature to treat the advanced LLC tumor animal model. The modified therapeutic ultrasound mode (Vflash) was delivered at a specified center frequency. The predefined ROI focused on the splenic region as the treatment area. Specific ultrasound parameters (MI: 0.98 to 1.03, acoustic pressure >2.0 MPa, frequency 5 MHz, PRF 500 Hz, and 1-s on/1-s off pulse pattern, and total treatment time 10 min) were performed on the spleen, which could impact the quantity of splenic immunosuppressive CECs. Acoustic pressure >2.0 MPa has reached the acoustic pressure amplitude for transient cavitation and lethal proration, disrupting the survival component (the membrane) of CECs. Our results showed that the frequency of splenic CECs was decreased while the proliferation of CD8^+^ T cells was increased after UTMD in the late-stage tumor animal model. The tumor growth curve showed that UTMD treatment alone could not inhibit tumor progression, while the combination of UTMD and PDL-1 blockade therapy could inhibit tumor growth. The antitumor immunity was increase by IFN-γ and TNF-α, as the frequencies of IFN-γ-producing CD8^+^ T cells (*p* = 0.0134) and CD4^+^ T cells (*p* = 0.0082) in the treated group were significantly increased compared with those in the control group. Additionally, the production of ROS and the frequency of TGF-β-producing CD11b^+^ cells were significantly reduced compared with those in the control group. Our study indicates that the biophysical effects produced by UTMD have great potential to enhance the acquired immunity in the advanced tumor, and the UTMD and PDL-1/PD-1 blockade combination therapy provides an option to overcome the challenges of immunotherapy that cancer patients are currently facing.

As the frequency of splenic CECs increased along with tumor progression (14), technically, the advanced tumor has more CECs in the spleen. We established a late-stage tumor model to determine whether UTMD could affect the frequency of CEC and lymphocytes in the spleen. The result suggested that CECs were reduced, and the T cells significantly increased, especially the CD8^+^ T cells. To testify to the safety of ultrasound treatment, we examined the spleen pathology at two different time points, instantly and 24 h after the treatment. H&E-dyed slice suggested no severe consequences on the splenocyte viability caused by UTMD. Based on the phenomenon that the immunoactivity was improved by ultrasound stimulation in the spleen of the late-stage tumor, we set out to investigate the long-term therapeutic effects generated by UTMD. To explore whether UTMD could affect tumor growth, we implemented the treatment three times and then measured the tumor size at different time points as described. The tumor growth curve line showed no difference between the two groups, which implied that the improved immunity generated by UTMD might not be enough to suppress the tumor development. However, UTMD-induced immunity improvement could be a prospective intervention to cancer-related infections in the advanced stage.

Since the UTMD monotherapy could not suppress the tumor growth, we assume that the combination of UTMD and PDL-1 blockade may lead to a better therapeutic effect. Emerging evidence suggests that immunotherapy drives new immune responses instead of reactivating the existing immune response in the TME. Yost et al. (3) reported that the PD-1 blockade therapy enhances antitumor response by recruiting novel T cells that may have recently entered the tumor instead of reinvigorating preexisting tumor-infiltrating lymphocytes. The latter may have limited immunocapacity. The T-cell response to checkpoint blockade derives from a distinct repertoire of T cell clones. Therefore, the key to improving the efficiency of blockade immunotherapy may lie in enriching T-cell proliferation outside the tumor environment. What we have discovered was exactly enhanced immunity beyond TME. During the activation and differentiation process of the CD8^+^ T cells, the CECs inhibited the CD8^+^ T-cell proliferation, which is the primer stage. The PD-1/PDL-1 pathway functioned in the cytotoxic T cell (28), which is the later stage in the TME. Based on our results, UTMD eliminated CECs from inhibiting CD8^+^ T proliferation and cells in the spleen, so more cytotoxic T cells in the peripheral circulation system could benefit from the immunotherapy.

Studies revealed that CEC expresses genes encoding immune checkpoint molecules (15, 29), so probably the immune checkpoint inhibitors could partially diminish the tumor-promoting effects of CECs. Meanwhile, the UTMD decreased the frequency of CECs, so the combination of UTMD and PDL-1 blockade may receive a better antitumor outcome than each other. We established a new protocol to determine whether UTMD combined with PDL-1 blockade therapy could inhibit tumor progression. The UTMD and PDL-1 blockade combination treatment was utilized compared with UTMD and PDL-1, respectively. The tumor growth curve showed that there were significant differences between the combination therapy group and the other groups. The RNA-sequencing analysis also showed that the gene expression profile of the combination therapy group was different from that of the control group. The heat map of the DEGs related to PD-1/PDL-1 pathway showed that the transcriptome profile of UTMD and PDL-1 blockade combination therapy-treated group was separate from that of the control group. These results verified our assumption that UTMD combined with PDL-1 blockade could enhance the antitumor effect. It is widely reported that the LLC tumors are resistant to checkpoint blockade, which is consistent with our result (30). The monotherapy is not enough. Optimized combinatorial strategies could extend the frontiers of anti-PD-1/PD-L1-based immunotherapy in lung cancer (31). More importantly, most of the current ultrasound-mediated cancer immunotherapy studies aimed to improve the immunity *in situ* or deliver genes and antigens by drug-loaded MBs or nanobubbles in the local tumor area. Our study uniquely focused on the systemic therapeutic effect enhanced by ultrasound. Our findings indicated that UTMD could improve the acquired immunity in the late-stage tumor, and UTMD-PDL-1 blockade combination therapy could inhibit tumor growth. It could be a prospective therapeutic approach for cancer patients.

To gain more insight into our study, we slightly modified the protocol and tested several related cytokine productions by flow cytometry. CECs suppress T-cell proliferation and the production of IFN-γ through direct cell-to-cell interaction (5). So we analyzed the productions of IFN-γ and compared them between the UTMD and PDL-1 blockade combination therapy and the control group. The results showed that both IFN-γ-producing CD8^+^ and CD4^+^ T cells were increased, but the MFI of IFN-γ only increased in CD8^+^ T cells. This phenomenon indicates that the key to UTMD-PDL-1 combination antitumor effects lies in IFN-γ, especially IFN-γ-producing CD8^+^ T cells. CEC also generates an immunosuppressive environment on releasing regulatory mediators such as ROS, TGF-β, and arginase-1, but our results showed the relative fractions of arginase-1-, TNF-α-, and TGF-β-producing CECs showed no differences between two groups. During the data analysis process, we noticed that a subset of cells, and the TNF-α-producing CD11b-negative cells, was particularly distinguishable. The cumulative data of the frequency of this population of cells showed no difference between the two groups. Nevertheless, it came to us that the CD11b cell surface marker might be the key to our study. Then we analyzed the frequency of the arginase-1-producing CD11b^−^ cells, which was significantly decreased, so was the frequency of TGF-β-producing CD11b^+^ cells, whereas there was no difference in arginase-1-producing CD11b^+^ cells. The CD11b is primarily expressed on granulocytes, monocytes/MCs, DCs, NK cells, and subsets of T and B cells. CEC co-expressing CD71^+^ and TER119^+^ in mice defines the nucleated erythrocytes as immature erythroid cells (32). We reasoned that UTMD combined with the PDL-1 blockade therapy might be mainly more affected by the erythrocytes. Thus, the proportion of arginase-1-producing CD11b^−^ cells had increased. In addition, CEC exerts immunosuppressive properties through TGF-β that promotes tumor growth and immune evasion (33, 34). Our results revealed that the combination therapy regulates the TGF-β production of CD11b^+^ cells to facilitate antitumor efficiency. The ROS production was also slightly reduced after the combination therapy. It corroborates with the putative mechanism that CEC produces ROS to suppress the immune response. The serum IL-2, produced by CD4^+^ and CD8^+^ T cells, is an important cytokine involved in the immune response and participates in antitumor effects (35). Unfortunately, no significant difference in serum IL-2 levels tested by ELISA was found between the two groups. To sum up, the combination of UTMD and PDL-1 blockade could inhibit tumor growth. The antitumor effect may enhance through IFN-γ. Loss of IFN-γ pathway genes leads to immunotherapy resistance (36), which means that the UTMD-PDL-1 combination therapy targeting CEC has great potential to enhance immunotherapy and could be a promising therapeutic strategy in oncology.

Obviously, there was some connection between CEC and UTMD, according to our results. However, the ultrasound–MB–cell interaction is transient and dynamic, which makes it hard to monitor *in vivo*. Besides, CEC differentiation is also a dynamic process, which makes it even harder. We deduced that the mechanism might involve the acoustic cavitation and perforation of the cell membrane. The CECs might be a target to mechanical stresses generated by UTMD. When administered intravenously, MBs were confined to the blood pool. Spleens present a particular type of microvessels absent in other organs, the venous sinuses (37). These microanatomical features make it a perfect cavitation site. Verified by intensity, there are two types of cavitation. High-intensity (>300 kPa) focused ultrasound generally disrupted the MB generated inertial cavitation, while low-intensity (<300 kPa) ultrasound generates stable cavitation. MI of 0.98~1.03 was set in this experiment, and the calculated acoustic pressure was over 2.0 MPa. Thus, the inertial cavitation played a dominant role. Cavitation activity of MBs creates microstreaming, shock waves, and microjets; and strains exert acoustic radiation forces, shear stress, and mechanical stress, causing pore formation or even disruption on the cell membrane (38–40). After exposure to MB-assisted acoustic cavitation, the wounded survival components (cell membrane and/or cytoskeleton) endangered the viability of CECs, so we observed a significant decrease of CECs. The CEC decreasing and the splenic lymphocyte proliferation increasing might occur independently or correlated. Since CECs inhibited CD8^+^ T-cell proliferation as reported (14), it was explicable that their percentage of splenocyte was inversely related, as our result has demonstrated. However, the ultrasound stimulation of the spleen could trigger the cholinergic anti-inflammatory pathway to modulate the T-cell and B-cell proliferation. Recently, researchers reported that non-invasive ultrasound stimulation targeting the spleen significantly reduces disease severity in a mouse model of inflammatory arthritis. Improvements are observed with specific parameters (1 MHz and 1-s on/5-s off pulse pattern) and acoustic pressure (e.g., ~350 kPa). Single-cell RNA sequencing of splenocytes revealed gene expression in T cells, and B cells are upregulated following ultrasound treatment in arthritic mice. Ultrasound stimulation alone might reduce the lymphocytes in the spleen. Therefore, in our study, the T- and B-cell proliferation may be caused by reducing CEC and/or the simulation of an anti-inflammatory pathway.

In summary, we innovatively introduced the immunosuppressive splenic CECs induced by cancer as a therapeutic target to enhance immune response and inhibit tumor growth by UTMD. The CEC was significantly reduced after UTMD. At the same time, the CD8^+^ T cells were increased without any pathological damages. Therefore, the UTMD treatment would be a valid intervention against cancer-related infections in the late-stage tumor. Meanwhile, the UTMD and PDL-1 combination therapy could inhibit tumor growth. Anemia treatment, targeting ineffective erythropoiesis and promoting CEC differentiation, are strategies reported against CEC, but they stay theoretical. Our study also has several limitations. We did not present evidence of CEC susceptibility to cavitation. The ultrasound–MB–cell interaction is transient and dynamic *in vivo*, which makes it hard to monitor. As for the mechanisms of our study, they also need more investigation. In our upcoming project, we plan to purify CEC *in vitro*, measure the membrane’s stiffness with an atomic force microscope, and verify some candidate genes’ function. In the meantime, PRF, duty cycle, pulse length, and acoustic pressure determine the cavitation intensity and form. A different combination of these parameters generates diverse biological effects; the parameters we use are effective ones, but the optimal combination of parameters merits further investigation. Despite these limitations, the treatments we provide are efficient and promising for cancer patients. In addition, ultrasound in medicine for therapeutic purposes has been applied by exploiting ultrasonic biological effects for years (41); the therapeutic approach we presented has great potential for clinical transformation.

## Data Availability Statement

The original contributions presented in the study are included in the article/supplementary material. Further inquiries can be directed to the corresponding author.

## Ethics Statement

The animal study was reviewed and approved by Laboratory Welfare and Ethics Committee of The Army Medical University.

## Author Contributions

ZL has designed the experiment, supervised the group, and revised the manuscript. XT has designed and performed the experiments, collected and analyzed the data, and wrote the manuscript. CY has participated in collecting and analyzing the data. YZ and NT participated in performing the experiments and collecting the data. YX has made suggestions, gave advice, and revised the manuscript. All authors contributed to the article and approved the submitted version.

## Funding

This work was supported by the National Natural Science Foundation of China (Grant Number: 2017YFC0107300).

## Conflict of Interest

The authors declare that the research was conducted in the absence of any commercial or financial relationships that could be construed as a potential conflict of interest.

## Publisher’s Note

All claims expressed in this article are solely those of the authors and do not necessarily represent those of their affiliated organizations, or those of the publisher, the editors and the reviewers. Any product that may be evaluated in this article, or claim that may be made by its manufacturer, is not guaranteed or endorsed by the publisher.
